# Valorization as a biofertilizer of an agricultural residue leachate: Metagenomic characterization and growth promotion test by PGPB in the forage plant *Medicago sativa* (alfalfa)

**DOI:** 10.3389/fmicb.2022.1048154

**Published:** 2022-12-22

**Authors:** Marina Robas Mora, Vanesa M. Fernández Pastrana, Agustín Probanza Lobo, Pedro A. Jiménez Gómez

**Affiliations:** Department of Pharmaceutical Science and Health, Montepríncipe Campus, CEU San Pablo University, Madrid, Spain

**Keywords:** alpha diversity, bacterial phytopathogens, biofertilizer, metagenomics, PGPB, waste recovery, germination

## Abstract

The abuse of chemical fertilizers in intensive agriculture has turned out in the contamination of ground and the soil on which they are applied. Likewise, the generation, storage, and destruction of plant residues from the agri-food industry poses a threat to the environment and human health. The current situation of growing demand for food implies the urgent need to find sustainable alternatives to chemical fertilizers and the management of agricultural waste. Valorization of this plant residue to produce natural biofertilizers using microbiological treatments is presented as a sustainable alternative. The microbial activity allows the transformation into simple molecules that are easily absorbed by plants, as well as the stimulation of plant growth. This double direct and indirect action induced significant increases against the variables of germination, viability, and biomass (dry weight). To guarantee biosafety, it is necessary to use new bio-technological tools, such as metagenomics, which allow the taxonomic analysis of microbial communities, detecting the absence of pathogens. In the present paper, a physicochemical and metagenomic characterization of a fertilizer obtained from agricultural plant waste valorization is carried out. Likewise, fertigation treatments were tested to which the Plant Growth Promoting Bacteria (PGPB) *Pseudomonas agronomica* and *Bacillus pretiosus* were added, both independently and in consortium. Metagenomic analysis has identified taxa belonging to the kingdoms Bacteria and Archaea; 10 phyla, 25 families, 32 genera and 34 species, none of them previously described as pathogenic. A 1/512 dilution of the fertilizer increased the germination rate of Medicago sativa (alfalfa) by 16% at 144 h, compared to the treatment without fertilizer. Both the fertilizer and the addition of PGPB in a double direct and indirect action induced significant increases against the variables of germination, viability, and biomass (dry weight). Therefore, the use of an agricultural residue is proposed, which after the addition of two new species is transformed into a biofertilizer that significantly induces plant growth in Mendicago sativa plants.

## Introduction

Eradicating hunger, feeding the future population and environmental conservation are the great challenges facing modern societies today. The application of chemical fertilizers in agriculture has resulted in eutrophication processes, an imbalance in soil physicochemical reactions and increased greenhouse gas emissions (Pirttilä et al., 2021). The latter is normal in the transformation of nutrients by plants. The increase in nutrients resulting from the application of chemical fertilizers leads to exponential increases in these emissions (Mikhailova, 2018). In this regard, the Food and Agriculture Organization of the United Nations (FAO), in its 2030 agenda, reports that intensive and short-sighted farming techniques have damaged ecosystems, causing the degradation of one-third of the planet’s soils. It also concludes that food production needs to increase by 50% to meet future food demand and advocates the use of environmentally, socially, and economically sustainable agricultural techniques (Colglazier, 2015).

Knowledge of techniques aimed at the recovery and new uses of plant residues is rising due to their contribution to the physiological and productive balance of soils. Plant remains serve as important points of microbial activity and constitute an elementary part of the preservation of edaphic carbon (Witzgall et al., 2021). Therefore, the use of plant residues as fertilizers can provide a sustainable solution to alleviate the depletion of soil nutrients caused by overexploitation (Chèneby et al., 2010). The recovery of plant waste destined for landfill relies on reclassifying it as having another useful purpose and making it a product with commercial and environmental benefits (Directive (EU) 2008/98/EC opinion of the Economic Committee; V. DIRECTIVA (UE), 2008).

Despite the known value in agriculture of fertilizers, other factors should be considered, such as the type of crop for which they are intended, agronomic practices, seasonality, host variants, location, bacterial strains, soil fertility and interaction with soil micro-biota. It is essential to know the efficacy of fertilizers; for this, biological germination tests are used. There are numerous germination test models, including those using alfalfa (*Medicago sativa* L.), which has been common in microbiota and agricultural ecology laboratories for more than 20 years (Stewart et al., 2001; Knežević et al., 2021). Alfalfa is, among the 23 fodder legumes, one of the most cultivated. Its annual production amounts to around 450 million tones worldwide. The main exporters are the United States (30%), Europe (25%) and Argentina (23%; Barros et al., 2019).

One of the main problems in the recovery of plant waste is its potential to carry microorganisms that are pathogenic to plants, the majority of which are bacteria. The use of a contaminated fertilizer can threaten crop growth, the environment and human health. To avoid the spread of pathogens and contaminants, it is essential to know their precise microbiological composition. The identification of some of these microorganisms is very complex and expensive, and some may even be undetectable because of the difficulty of their cultivation or because of their low concentrations when using more conventional techniques (Blessing and Oluranti, 2022). Metagenomics enables the rapid and accurate identification of many micro-organisms, including those that are either uncultivable or underrepresented that either pose a risk or that provide value due to their ability to promote plant growth. PGP bacteria can act by increasing the cycle of nutrients, which is why their use is progressively increasing in agriculture as substitutes for chemical fertilizers, herbicides, antibiotics and pesticides (Wang et al., 2021). Numerous studies have been carried out to confirm this promising practice where *Bacillus* spp. and Pseudomonas spp. They have been proposed as good promoters of plant growth with great resistance to abiotic stress of crops, among other qualities (Katsenios et al., 2022).

For the above reasons, metagenomics is postulated as a suitable technique that allows for more thorough characterization of the composition of the microbial taxa of a certain matrix of a residue/leachate, identification of possible bacterial phytopathogens and the biological interpretation of fertilizer behavior (Blessing and Oluranti, 2022).

Given the impact of the intensive use of chemical fertilizers in cultivation on the environment and human health, it is necessary to increase the recovery of waste as a sustainable measure. To contribute to the knowledge of the techniques and measures that favor the sustainable exploitation of the soil, two objectives are proposed: (i) carry out physicochemical and metagenomic analyzes of ORGAON^®^ for the identification of different taxa, whose results can confirm the safety of a fertilizer through the verification the absence of bacterial plant pathogens; (ii) evaluate the effect on germination, growth promotion and viability of Medicago sativa treated with a biofertilizer composed of the extract of a recovered plant leachate and the addition of the PGPB *Pseudomonas agronomica*, *Bacillus pretiusos* and its consortium.

## Results

### Agricultural leachate residue analysis

To comply with Annex IV statements of Regulation (EC) 2003/2003 and Annex VI of RD 506/2013 (2013), which establishes the physicochemical criteria for plant residues, physicochemical analysis of the ORGAON^®^ fertilizer was done. The results are shown in Table 1.

**Table 1:** Physicochemical composition of ORGAON^®^ valorized leachate.

Nutrients	s.m.o. % (*w*/*w*)	s.m.s. % (*w*/*w*)	s.m.o. % (*w*/*w*)	s.m.s. % (*w*/*w*)
Ashes	4.72 ± 1.11	80.60 ± 4.85	4.92 ± 1.02	84.00 ± 3.72
Total organic matter	1.13 ± 0.06	19.40 ± 3.20	1.18 ± 0.72	20.20 ± 2.22
Total organic C	0.66 ± 0.20	11.23 ± 3.33	0.68 ± 0.11	11.69 ± 2.21
Total humic extract	3.94 ± 0.12	67.20 ± 5.02	4.10 ± 1.01	70.00 ± 3.42
Humic acids	<0.10 ± 0.02	<1.71 ± 0.78	<0.10 ± 0.05	<1.78 ± 0.85
Fulvic acids	3.94 ± 1.09	67.20 ± 3.27	4.10 ± 1.00	70.00 ± 2.78
Nitrogen	0.10 ± 0.03	1.79 ± 0.09	0.11 ± 0.02	1.86 ± 0.99
Ammonium	0.02 ± 0.01	0.41 ± 0.19	0.02 ± 0.01	0.43 ± 0.03
Nitric N	(22.6×10^−3^) ± 0.3	(38.6×10^−3^) ± 0.5	(23.50×10^−3^) ± 0.25	(40.20×10^−3^) ± 0.4
Urea N	<0.10 ± 0.08	<1.71 ± 0.76	<0.10 ± <0.01	<1.78 ± 0.21
Organic N	<0.10 ± 0.01	<1.71 ± 0.71	<0.10 ± <0.01	<1.78 ± 0.09
Inorganic P	2.78 ± 1.91	47.26 ± 4.23	2.89 ± 0.82	49.48 ± 2.12
Total K (K_2_O)	2.58 ± 1.52	44.00 ± 3.01	2.69 ± 0.97	45.90 ± 2.01

s.m.o.: compared to the original sample; s.m.s.: compared to dry sample; *w*/*w*: weight/weight; (*w*/*v*): weight/volume; n.d.: not detected (quantification limit < 0.5 mg.kg^−1^ s.m.o). Results are followed ± SD (*n* = 3).

The results meet the regulatory criteria applicable to fertilizer products with residues and other organic components (Hedo, 2013).

### Metagenomic analysis: Sequencing, clustering and taxonomic assignment

Metagenomic analysis was carried out using different processes. As a result of the bioinformatic analysis, metrics and indices were obtained that allowed us to interpret the quality of the processing as well as the composition and taxonomic abundances of the microbial community of the sample. These parameters are shown in Table 2.

**Table 2:** Sequencing, clustering, and taxonomic assignment process statistics.

Process	Metrics and Indices	Results
Sequencing clustering	Data size (Mbp)	74 ± 3
	Total raw readings (No.)	247.33 ± 8.3
	Average length of readings (bp) (SD)	300.20 ± 25.3
	Q30 Score (%)	67 ± 3
	Reads merged totals (No.)	188.76 ± 18.5
	Merging (%)	76.30 ± 5.4
	ZOTU totals (No.)	96 ± 16
	Total effective readings (No.)	140.82 ± 7.2
	Singleton totals (No.)	0.00
	Good coverage (%)	100
Taxa	Kingdom (%)	100
	Phylum (%)	100
	Class (%)	99.50
	Order (%)	98.70
	Family (%)	90.00 ± 1.90
	Gender (%)	88.30 ± 2.01
	Species (%)	78.60 ± 5.29

Good coverage: value representing how well the sequences obtained represent complete populations. Results are followed ± SD (*n* = 3).

**Figure 1:**
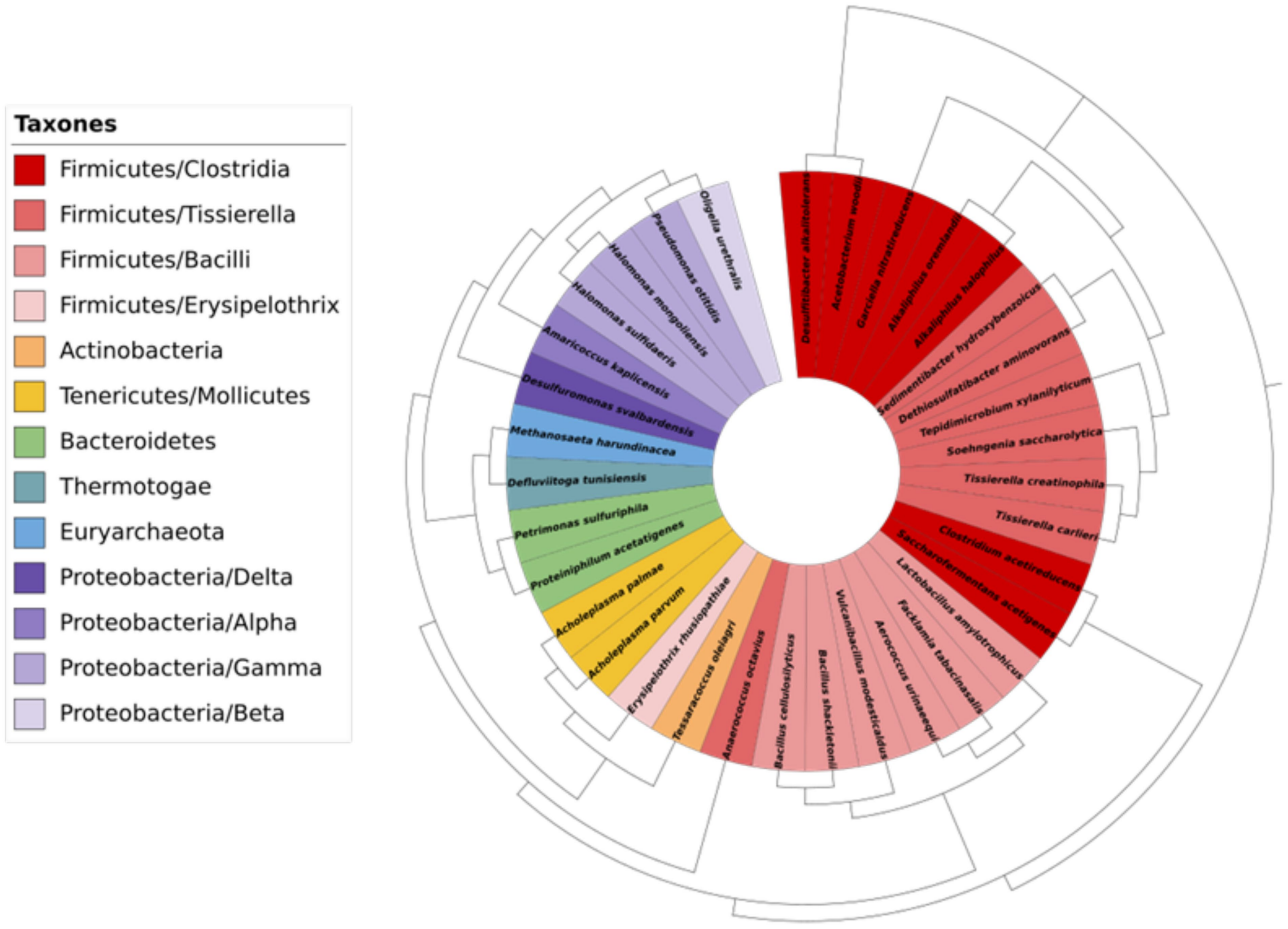
Graphic representation of the taxonomic diversity of ORGAON^®^.

The following taxonomic categories were identified: 2 kingdoms, 10 phyla, 25 families, 32 genera and 34 species. An observed relative abundance of bacterial population of 99.62% and archaeobacteria population of 0.38% was found. The main bacterial taxa found in the samples, above 1% relative abundance, were Firmicutes (38.32 ± 0.25%), Proteobacteria (29.09% ± 0.16%) and Bacteroidetes (26.73% ± 0.17%). The taxonomic grouping of the taxa identified at division and order level in ORGAON^®^ fertilizer is shown in Figure 1. The complete list of taxa and their relative abundance are collected in Supplementary Table S1.

No reference has been found in the scientific literature on the phyto-pathogenic potential of any of the identified species.

### Alpha diversity indices

Metrics resulting from the alpha diversity indices reflect the structure of a community with respect to its richness (number of taxonomic groups that make up the community) or evenness (distribution of groups with respect to their abundance), or both (Willis, 2019). The analyzed indices and their ranges of interpretation are shown in the Table 3.

**Table 3:** Alpha diversity indices of ORGAON^®^ fertilizer.

Alpha diversity indices	ORGAON^®^
Shannon index (H)	3.64 ± 0.25
Simpson’s index (D)	0.08 ± 0.01
Pielou index (J)	0.80 ± 0.09
Phylogenetic diversity index (PD)	10.34 ± 1.20

Results are followed ± SD (*n* = 3).

### Optimum testing conditions for ORGAON^®^ fertilizer

**Figure 2:**
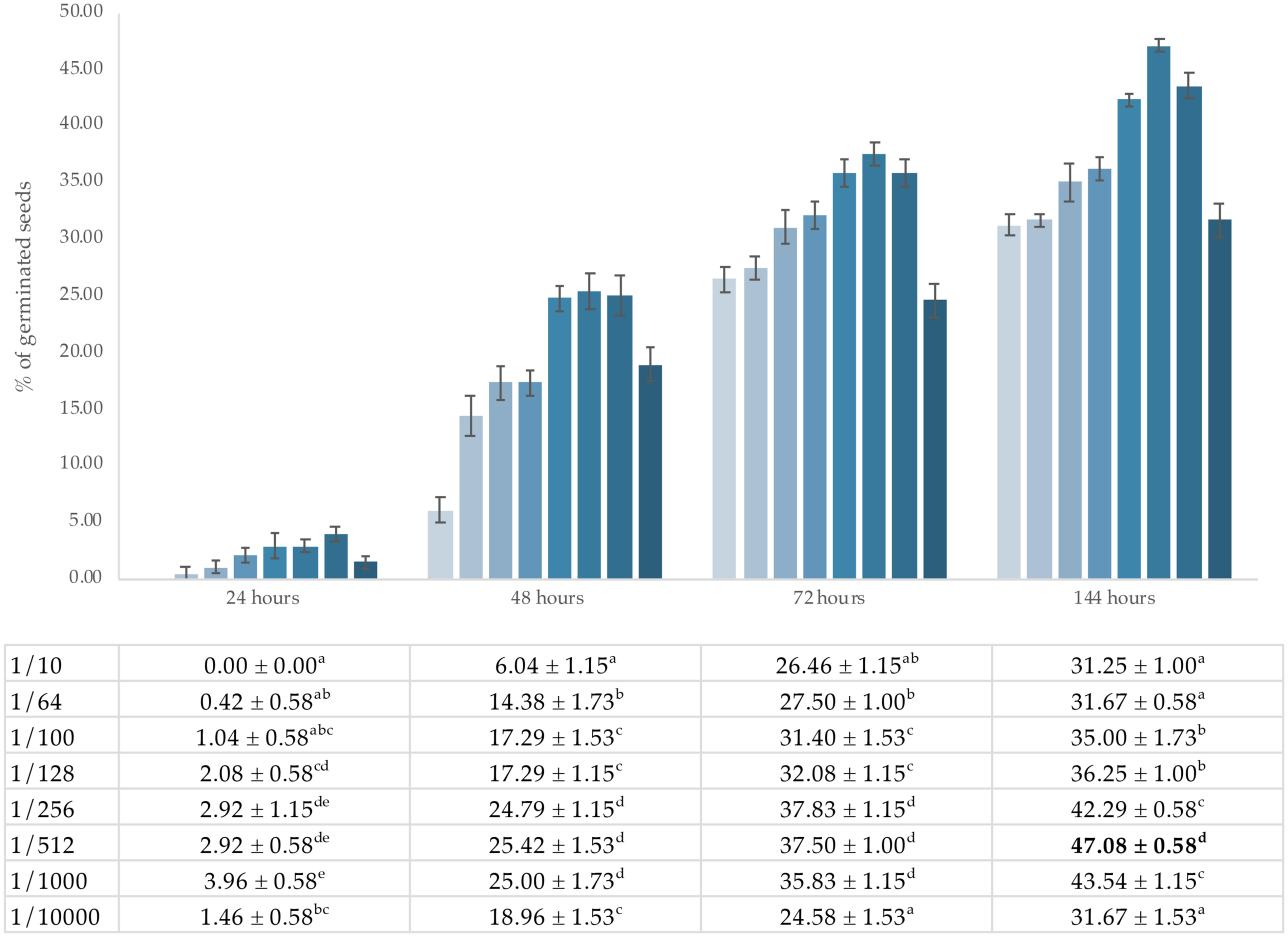
Percentage of germinated seeds after 24, 48, 72, and 144 h for each dilution. All values are the mean of three replicates. Results are followed ± SD (*n* = 3). The data with identical superscript letters indicate that the mean values are not significantly different.

To analyze the efficacy of the fertilizer, its effect on the germination of the *Medicago sativa* L. plant model was tested. Figure 2 shows the results of the dilution tuning (*v*/*v*) of the ORGAON^®^ fertilizer in mineral water. Each condition was tested in triplicate and the percentage of mean values is shown in the graph.

The maximum germination rate was reached at dilution 1/512 (*v*_Fert ORGAON_^®^/*v*_mineral water_) 144 h after sowing. Under these conditions, the germination rate was 46.88%.

**Figure 3:**
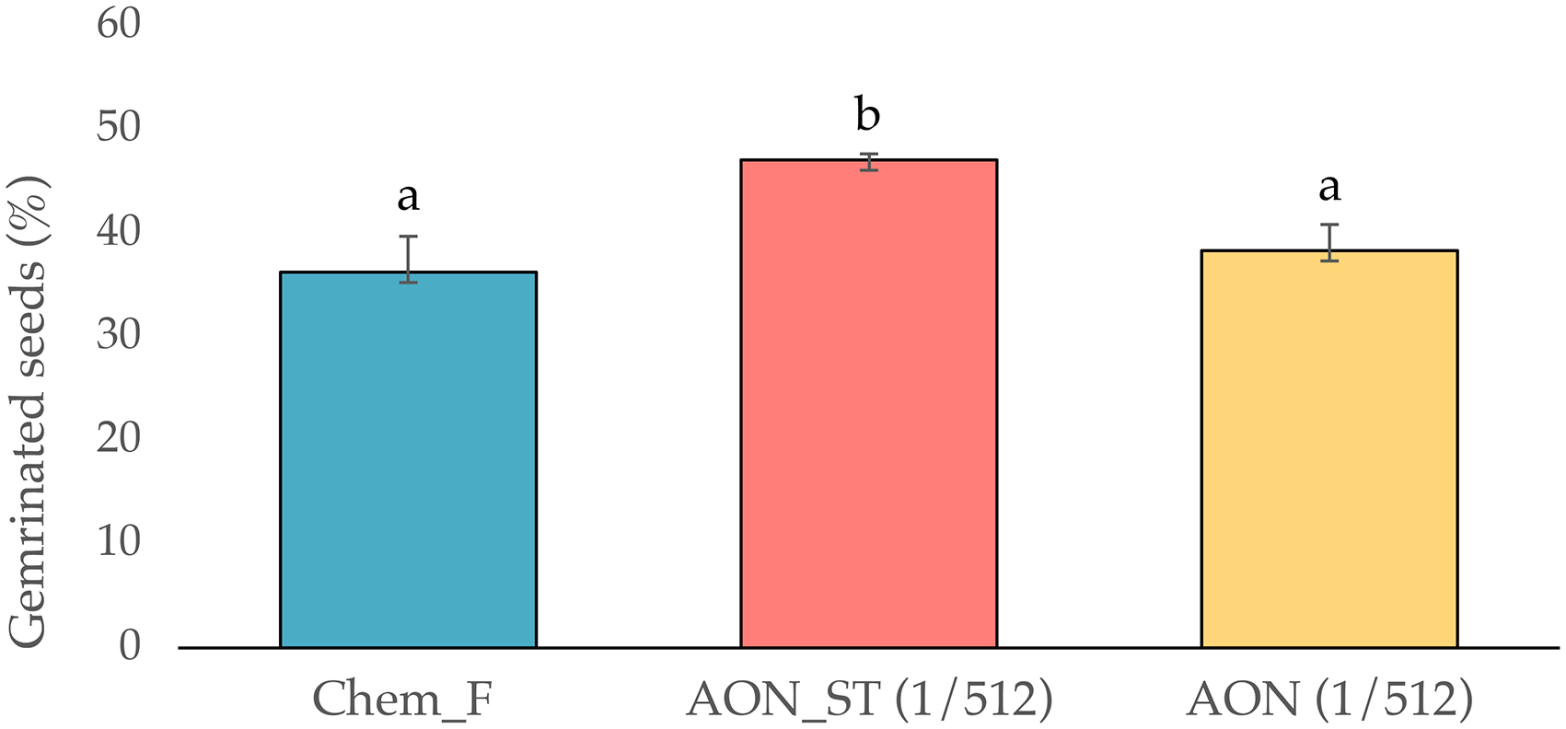
Germinated seeds (%) under different fertigation conditions. Results are followed ± SD (*n* = 3). Data with identical superscript letters indicate that the mean values are not significantly different.s

The comparison between the germination rate in the described conditions versus the traditional treatment with Chem-F irrigation is shown in Figure 3.

### Germination trial

**Figure 4:**
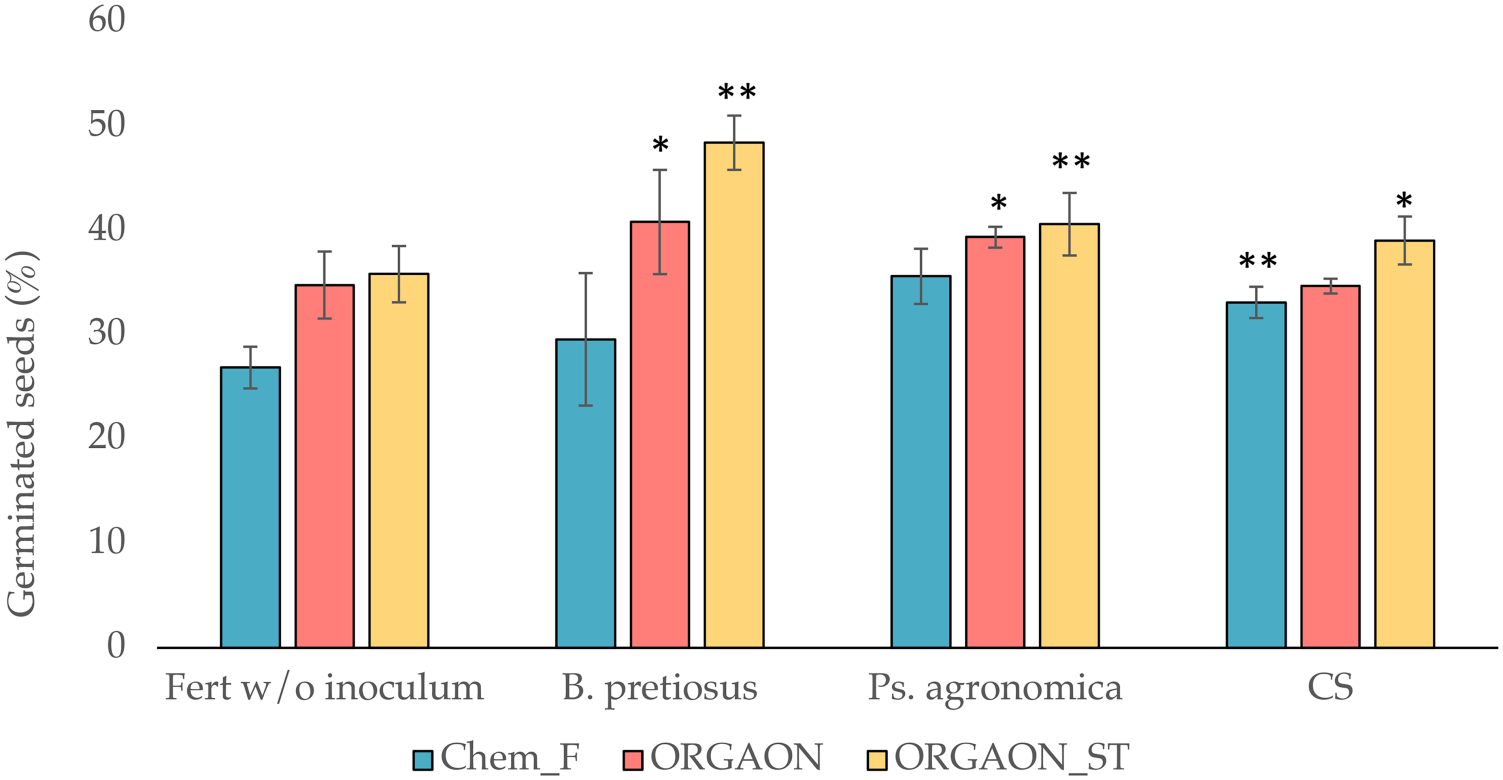
Germinated seeds (%) under the different fertigation conditions. ORGAON^®^ and ORGAON^®^_ST at the experimental concentration 1/512. Asterisks represent treatments with significant differences in germination compared to their control without inoculum and traditional fertilization (Chem-F; ^*^p < 0.05; ^**^p < 0.01).

To know the effect of the addition of PGPB *Pseudomonas agronomica*, *Bacillus pretiosus* and its consortium in the matrix of ORGAON^®^ and ORGAON^®^_ST fertilizers, a germination assay was carried out with *Medicago sativa* var. Aragon. The corresponding Chem-F controls were used, to which the same strains and consortium were added. All the measurements collected in Figure 4 correspond to the average of three replicates per treatment.

ORGAON^®^ and ORGAON^®^_ST treatments without PGPB additives show higher germination rates than Chem-F. These increases are not significant. However, the addition of the *Pseudomonas agronomica* and *Bacillus pretiosus* to organic fertilizers achieve significantly higher germination rates with respect to their controls. The same happens with the treatment of Chem-F added with *Pseudomonas agronomica* and the bacterial consortium with respect to its control without bacterial inoculum.

In the trials of plants fertigated with ORGAON^®^ and ORGAON^®^_ST, the greatest differential in the germination rate is observed in those treatments added with the *Bacillus pretiosus* strain. When this strain is tested in an ORGAON^®^ matrix, the differential increase compared to the uninoculated fertilizer is 17.37% and increases to 35.17% when the bacteria is tested in an ORGAON^®^_ST matrix. The addition of *Pseudmonas agronomica* strain to the ORGAON^®^ and ORGAON^®^_ST matrices also induced an increase in germination, with 13.37 and 17.13%, respectively. Likewise, the consortium of both induced a 9.01% increase in germination in the treatment with ORGAON^®^_ST with respect to the chemical fertilizer Chem-F added with the same strains.

### Plant growth assay

**Figure 5:**
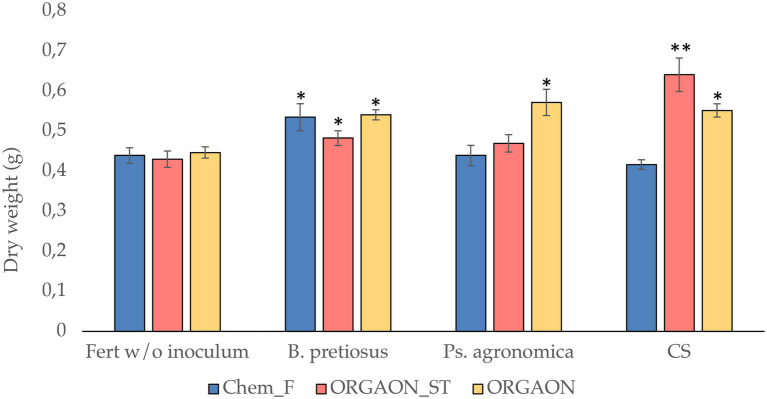
Dry weight (g) of seedlings after 6 weeks of growth. The asterisks represent significant differences with respect to the control without inoculum and traditional fertilization (Chem-F; ^*^p < 0.05; ^**^p < 0.001).

A growth assay was performed, reproducing the irrigation conditions with Chem-F, ORGAON^®^ and ORGAON^®^_ST in the presence and absence of independent and consortiated PGPB. Figure 5 shows the variation of the dry weight of the plants once the trial is over.

A significant increase in dry weight is observed in those plants treated with ORGAON^®^ added with *Pseudomonas agronómica*, *Bacillus pretiosus* and their consortium, compared to any fertilizer control without bacterial addition.

### Seedling viability test

**Figure 6:**
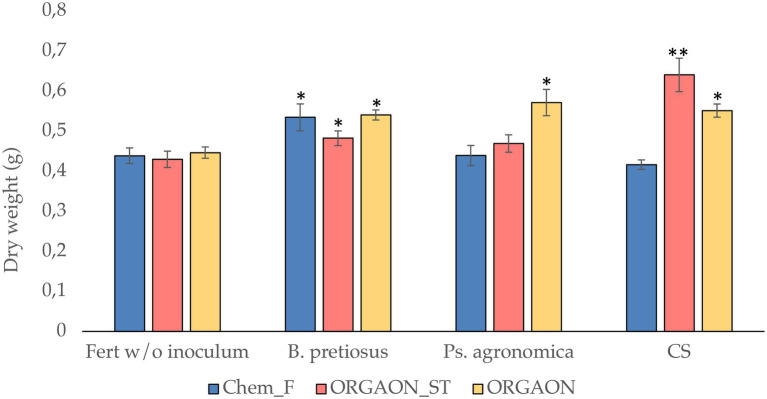
Seed viability (No.) at 6 weeks of growth. Asterisks represent treatments with significant differences in germination compared to their control without inoculum and traditional fertilization (Chem_F; ^*^p < 0.05; ^**^p < 0.01).

Seedling viability was analyzed 6 weeks after germination. Figure 6 shows the results of the count of viable plants.

The addition of *Bacillus pretiosus* to any fertilizer significantly favors the viability of the treated plants. Said phytoprotection is remarkably superior when the bacterium is in the presence of the ORGAON^®^ fertilizer, both in its natural formulation and in its sterilized form. The PGPB *Pseudomonas agronomica* and the consortium treatment of both show similar results when tested in the ORGAON^®^ matrix.

## Discussion

The use of waste as a raw material for sustainable industrial and agronomic processes is a priority. It is necessary to ensure compliance with the nutrient concentration requirements of current environmental regulations. At the physicochemical level, it is essential that the concentration of toxic substances is minimal (Hedo, 2013). It is also important to characterize the microbiological composition to guarantee the safety and security of the product prior to mass agronomic application. The ORGAON^®^ dilution factor that proved to have a better effect on germination was 1/512 (on mineral water). For this concentration to guarantee the efficacy of the organic fertilizer, the standardization of the physicochemical variables must be guaranteed (Table 1). it must be taken into account to reproduce the same dilution factor and optimize its use in plant growth and development (Phibunwatthanawong and Riddech, 2019).

Metagenomic analysis (mNGS) is based on unbiased sampling. In the present study, this technique achieved taxa identification at the species level for 78.6% of the observed relative abundance in the sample. This method allows the identification of expected, un-expected and unknown microorganisms. Likewise, it allows obtaining quantitative or semiquantitative data on the absolute and relative concentrations of each identified taxon (Gu et al., 2019). For these reasons, it is widely used as a routine method in the analysis of complex environmental samples (Franssen et al., 1784; Borthong et al., 2018; Taş et al., 2021). Authors such as Suttner et al. consider metagenomics to be a useful tool for the identification of microorganisms, risk assessment and the minimization of public health hazards (Suttner et al., 2020).

The bacterial communities present in complex mixtures, such as fertilizers, can be extremely heterogeneous. In the ORGAON^®^ fertilizer studied, the main isolates belong to the phyla Proteobacteria, Actinobacteria, Bacteroidetes and Firmicutes, accounting for 94.38% of the heterogeneity. These results agree with metagenomic studies by other authors, who described these as the predominant taxa (Clúa et al., 2018). Alphaproteobacteria class has been referred to in the use of fertilizers. Its abundance is dependent on the concentration of nutrients, increasing in the presence of phosphate due to their ability to solubilize it and decreasing with increased nitrogen. This class accounts for 25.81% of the sample (Dai et al., 2020). The Clostridiaceae family is identified as one of the groups in which there is biotechnological interest for the development of organic nitrogen fertilizers. This family is present in 3.34% of the sample (Ward et al., 2018). Other authors note the presence of *Clostridiales* (30.33%) in fertilizers; this taxonomic order is known to have the ability to reduce ferric iron (Fe^3+^), and most of its species can fix nitrogen [II]. When considering a lower taxonomic resolution, no references in the scientific literature on natural fertilizers have been found regarding the predominance of those taxa identified, in the most abundant sample. However, references to some less preponderant taxa have been identified in studies on the selective gradient of micro-biological diversity along the root–plant continuum, which confirm the presence and abundance of the genus Bacillus. In these samples, species belonging to this genus were present in 3.23%; likewise, the order *Pseudomonadales* corresponded to 0.66% of the relative microbial abundance of the ORGAON^®^ fertilizer (Clúa et al., 2018).

One of the dangers in the use of natural fertilizers whose origin is a plant extract is the potential transmission of bacterial phytopathogens to the target crops. For this reason, it is necessary to detect these pathogens in samples to ensure environmental biosafety (Franssen et al., 1784; Borthong et al., 2018). Discrimination of the presence or absence of bacterial phytopathogens requires identification at the species, subspecies or pathotype level, as appropriate (Franssen et al., 1784). In taxonomic identification studies of isolated culturable bacteria, the identity index with which the 16S sequence of the bacterium is aligned should not be less than 99%. This ensures accurate identification of the isolated strain. In metagenomics analysis, there is a risk of not detecting a plant pathogen if it is found in very low proportions (Miller et al., 2013; Andrusch et al., 2018) or when the analysis involves unspecific sequences (Borthong et al., 2018). To avoid this, the identity index threshold is reduced. In this way, the detection of incomplete or minority sequences could be used in alignments for determining the presence of the corresponding organisms. In line with what was observed in another study (Hao and Chen, 2012), we reduced the identity index requirement to 90% in the present work. For this reason, any sequence like the identification of a phytopathogen will allow its detection. In the same sense, surveillance based on nontargeted sequencing is being promoted as part of several European initiatives—the European Epidemiological Network (Epi-NET) and the EU Action Plan—intending to include the development of integrated surveillance systems (Duarte et al., 2020). Having applied this criterion, in the present work, the results indicate that the ORGAON^®^ sample is absent of bacterial phytopathogens.

ORGAON^®^ fertilizer combines plant residues from various crops, resulting in a rich liquid fraction that carries PGPB organisms. The alpha diversity metrics of all calculated indices shows a wide diversity. These indices are presented as good indicators for monitoring ecological or phenological samples.

The Shannon index (Dai et al., 2020) is commonly used to characterize the diversity of species in a community and explains both the abundance and evenness of the species present. The analysis of the ORGAON^®^ fertilizer yields a value of 3.64 on the Shannon index, which indicates high microbial diversity. This measure considers the proportion of each species in a studied ecosystem, thus providing a good representation of diversity, beyond a simple count of the number of species (Konopiński, 2020) [50]. However, this index has limitations in the weighting of ZOTUs (Hill et al., 2003). To overcome this, other alpha diversity indices were used. The Pielou (J) index (0–1) applied to the ORGAON^®^ fertilizer sample is 0.80, which corroborates the high diversity estimated by the Shannon index. Likewise, Simpson’s index (D) measures the proportion of observed diversity in relation to the maximum expected diversity. Values close to 1 correspond to situations where all species are equally abundant, and 0 indicates the degree of maximum inequality (Soler et al., 2012). The result obtained for this index reflects a result consistent with the Shannon and Pielou index. Finally, the phylogenetic diversity index (PD) uses phylogenetic patterns of evolutionary diversification to estimate the evolutionary diversity of a community. The result of 10.34 indicates a high phylogenetic diversity, and it can be interpreted that the ORGAON^®^ fertilizer matrix can host taxonomically and functionally very diverse organisms. In the Earth Microbiome Project (EMP), more than 11,000 samples from different ecosystems were analyzed, and the alpha, beta and gamma diversities of bacterial assemblages were calculated. Agricultural soils were found to be one of the richest systems. The EMP has continued its analysis, with more than 27,000 samples from different geographical areas. A recent review of the project pointed out that there is a higher diversity of free-living bacteria than those associated with a host, so that a soil or sediment sample would reach a higher alpha diversity than a gut micro-biome. The results obtained in the ORGAON^®^ metrics agree with current study, in which plant residues and agricultural land provide an optimal environment for microbial richness (Walters and Martiny, 2020). From the above, it can be interpreted that the ORGAON^®^ fertilizer analyzed in the present work respects the diversity of edaphic microbial communities.

Germination data show that the maximum rate was reached at dilution 1/512 (*v*_Fert ORGAON_^®^/*v*_mineral water_) 144 h after sowing. Under these conditions, the germination rate was 46.88%. This increase affects both the germination rate and the viability of *Medicago sativa* seed. These effects depend on the concentration (*v*/*v*) of the fertilizer, with the optimum concentration being 1/512 dilution. The average germination time is 2–4 days in a temperature range of 22 ± 2° C, as established by Benabderrahim et al. (2011). The number of germinated seeds was 46%, a percentage that falls within the expected range presented in other studies (Benabderrahim et al., 2011). However, fertilizer concentrations significantly affect the germination percentage and need to be adjusted to optimum levels. The percentage of germinated seeds is in accordance with results described by other authors, who mention that organic fertilizers are nutritional supplements that improve the yield and growth of agricultural crops (Gómez-Álvarez et al., 2008; Li et al., 2020). The reason for this is the increase in the availability of nutrients that are not easily assimilated by the plant under natural conditions and thus improve soil degradation.

The addition of *Pseudomonas agronomica* and *Bacillus pretiosus* to ORGAON^®^ and ORGAON^®^_ST induce significantly higher germination rates with respect to the fertilizer controls without additives with PGPB. The same happens with the treatment of Chem-F added with *Pseudomonas agronomica* and the bacterial consortium with respect to its control without bacterial inoculum. This fact evidences the promoting effect of both PGPB on the germination of *Medicago sativa* seeds regardless of the fertilizer matrix that contains them. The addition of PGPB bacteria facilitates the nutrient acquisition in plants, increasing the germination percentage. The fact that it is a liquid fertilizer means that it is distributed in a more homogeneous way, being available in similar concentrations for the entire plant (Scialabba and Hattam, 2002).

Likewise, the plants treated with ORGAON^®^ show significant differences for dry weight in any of their supplemented treatments, both with *Pseudomonas agronomica*, *Bacillus pretiosus* as its consortium. Similar results are obtained when the tested fertilizer is ORGAON^®^_ST. This fact reinforces the explanation of the beneficial effect of the transformation of the organic fertilizer by the metabolic action of PGPB, which gives rise to a better availability of nutrients for the plant (Gómez-Álvarez et al., 2008). Therefore, the bacteria that are added to organic fertilizers improve the conditions of the plant, both directly through the synthesis of biomolecules with PGP activity, and indirectly through the prolonged provision for the plant of easily adsorbed transformed nutrients. This transformation optimizes the use of nutrients, which leads us to think that their use is more efficient and sustainable (Li et al., 2020).

After germination, seedling viability can be compromised by numerous environmental factors. Among the microbial biotic factors that can compromise viability are the colonization of phytopathogens (Kumar and Verma, 2018) or the loss of competitiveness due to the absence of beneficial bacteria for the plant (Cheng et al., 2020). The edaphic and rhizospheric colonization of PGPB hinders the implantation and development of other microorganisms that could behave as bacterial phytopathogens (Parray et al., 2016; Doty, 2018; Gouda et al., 2018). In this sense, the viability of the seedlings is significantly higher when the treatment consists of the contribution of *Bacillus pretiosus*, *Pseudomonas agronomica* and their consortium in the presence of the ORGAON^®^ fertilizer in its natural formulation. The HCN synthesis capacity of both *Pseudomonas agronomic* as of *Bacillus pretiosus* (Robas Mora et al., 2022) minimizes the possibility of root colonization of phytopathogens. Thus, the presence of PGPB microorganisms in this type of fertilizer could have a phytoprotective effect (Backer et al., 2018; Naeem et al., 2018; Blake et al., 2021). Thus, the joint effect of all these factors can explain the significant difference in viability of the plants treated together with ORGAON^®^ added with the PGPB under study.

Prior to the use of these fertilizers, biosafety must be guaranteed. In this way, such fertilizers can be a sustainable alternative for organic. Liquid biofertilizers are associated with higher crop yields and therefore lower costs (Ye et al., 2020). Previous studies have shown that inoculation with PGPB organisms on crops treated with chemical fertilizers increases nutrient use efficiency. This efficiency is measured by considering plant growth. Likewise, PGPB organisms reduce the need for chemical fertilizers by 50% without causing yield loss. According to the studied parameters, the addition of these fertilizers stimulates germination, promotes plant growth, and can have a positive effect on the edaphic microbial community (Hayat et al., 2010; Good and Beatty, 2011; Da Costa et al., 2013).

For all these reasons, it can be concluded that the fertilizer ORGAON^®^ can be used under biosafety conditions since the metagenomic study of the *16S rRNA* amplicons reveals the absence of pathogens and phytopathogens. Experimentally, the maximum germination rate of *Medicago sativa* seeds (46.88%) was reached at the 1/512 dilution (*v*_Fert ORGAON_^®^/*v*_mineral water_) 144 h after inoculation. Likewise, irrigation with ORGAON^®^ fertilizer under these conditions results in a significant increase in germination, 16% more than traditional irrigation with inorganic fertilizers (Chem-F). Finally, the incorporation of *Pseudomonas agronomica* and *Bacillus pretiosus* strains in ORGAON^®^ (biofertilizer) promotes germination, total biomass, and viability of *Medicago sativa* var. Aragon.

## Materials and methods

### Sample collection

The collection of plant waste is carried out over 30,000 hectares of greenhouses, from which two million tons are collected, including stems and leaves, and discarding fruits and complete plants (Biaqui, Almería, Spain). This land extends over different farms in the region of Dalías, province of Almería, Spain (36°49′17″N 2°52′13″W). The average annual temperature is 18°C, and the annual sunshine duration is 3,305 h. These remains must be compulsorily collected and treated for their elimination or revaluation. During composting, a liquid fraction (leachate) is produced, composed mainly of the water from the plants that are being composted. The liquid fraction is subjected to a stabilization process once collected from the composter and then stored (ORGAON^®^). To evaluate the leachates, a 1 l sample was collected in triplicate. The sample was deposited into a sterile container and transported and stored at 4°C for immediate processing. Each sample was made up of four 250 ml subsamples, taken from different depths of the collection tank (5 m^3^), where the leachate is collected and stored under aerated conditions.

### Fertilizer composition

#### ORGAON^®^ fertilizer

Table 4 shows the physicochemical analysis carried out on the samples and the respective methods used in accordance with Annex IV to Regulation (EC) 2003/2003 and Annex VI to RD 506/2013 of 28 June, which specify the physicochemical criteria for plant residues.

**Table 4:** PTA-FQ: physicochemical testing of EC fertilizers and other fertilizer products.

Determinations	Methodology
Moisture	PTA-FQ-024, drying at 105°C
Dry matter	PTA-FQ-024, drying at 105°C
Density at 20°C	Gravimetry
pH	PTA-FQ-004, pH meter, based on UNE-EN13037
Electrical conductivity at 25°C	PTA-FQ-005, drive meter, based on UNEEN 13038
**Nutrients**	**Methodology**
Ashes	PTA-FQ-022, 540°C calcination, based on UNE-EN 13039
Total organic matter	PTA-FQ-022, calcination, based on UNE-EN 13039
Total organic carbon	PTA-FQ-022, mathematical calculation
Total humic extract	PTA-FQ-014, dichromate oxidation, meth. 4 R.D. 1,110/1991
Humic acids	PTA-FQ-014, dichromate oxidation, meth. 4 R.D. 1,110/1991
Fulvic acids	PTA-FQ-014, dichromate oxidation, meth. 4 R.D. 1,110/1991
Nitrogen	PTA-FQ-036, Dumas, based on UNE-EN 13654–2
Ammonium nitrogen N	PTA-FQ-053, ion chromatography, based on UNE-EN 14911
Nitric nitrogen N	PTA-FQ-006, ion chromatography, based on UNE-EN 10304–1
Urea nitrogen N	PTA-FQ-041, HPLC-UV, based on UNE-EN ISO 19746
Organic nitrogen N	PTA-FQ/020, mathematical calculation, based on R.D. 1,110/1991 annex Num. 4
Total potassium K_2_O	PTA-FQ-027, ICP-AES, based on UNE-EN 16963
**Heavy metals**	**Methodology**
Total cadmium Cd	PTA-FQ-027, ICP-AES, based on UNE-EN 16963
Total copper Cu	PTA-FQ-027, ICP-AES, based on UNE-EN 16963
Total chromium Cr	PTA-FQ-027, ICP-AES, based on UNE-EN 16963
Total mercury Hg	PTA-FQ-027, ICP-AES, based on UNE-EN16963
Total nickel Ni	PTA-FQ-027, ICP-AES, based on UNE-EN16963
Total lead Pb	PTA-FQ-034, ICP-AES, based on UNE-EN16963
Chromium VI Cr (VI)	PTA-FQ-034, HPLC-UV, based on UNE-EN16318
Total zinc Zn	PTA-FQ-027, ICP-AES, based on UNE-EN16963

#### ORGAON^®^_ST fertilizer

The synthesis of ORGAON_ST^®^ consisted of exposing a thin film of OR-GAON^®^ to UV light for 15 min, to avoid substantial changes in its physicochemical composition.

#### Chemical-fertilizer (Chem-F)

PK complex fertilizer is rich in phosphorus and potassium, with magnesium and Sulphur. Low in chloride. Composition: nitrogen 0% + phosphorus 7% + potassium 14% + sulfur 25%. Formulation: (K2HPO4 5%); (K2SO4 14%); (MgSO₄ 2.5%).

### Metagenomic analysis

#### Extraction of DNA and PCR

DNA was extracted from 200 μl of undiluted sample (prepared in Section 2.1) using the All Prep Power Viral RNA/DNA (Qiagen, Hilden, Germany) kit, according to the manufacturer’s instructions. A total of 3 ng of DNA (measured by Picogreen, Thermofisher) was used for the first PCR, with the enzyme Q5Ǐ Hot Start High-Fidelity DNA Polymerase (New England Biolabs), in a volume of 25 μl and with a concentration of primers of 100 nM. The primers used amplified the 16S rRNA V3–V4 regions and added extra sequences, on which the second PCR was performed:

CS1-341F 5′-ACACTGACGACATGGTTCTACACCTACGGGNGGCWGCAG-3′.
CS2-805R ′-TACGGTAGCAGAGACTTGGTCTGACTACHVGGGTATCTAATCC-3′.

The CS1 and CS2 sequences are indicated in bold. The cycling of the first PCR was as follows: 1 × 98°C for 30 s; 20 × (98°C for 10 s, 50°C for 20 s and 72°C for 20 s) and 1 × 72°C for 2 min.

The second PCR was in a volume of 20 μl over 1 μl of the amplification product of the first 1/50 diluted PCR. For this purpose, the enzyme Q5Ǐ Hot Start High-Fidelity DNA Polymerase (New England Biolabs) was used in the presence of 400 nM of the primers 5′-AATGATACGGCGACCACCGAGATCTACACTGACATGGTTCTACA-3′ and 5′-CAAGCAGAAGACGGCATACGAGAT-[10-barcode-nucleotides]-TACGGTAGCAGAGACGGTCT-3′ from the Fluidigm house (see Array Barcode Li-brary for Illumina Sequencers). The cycling conditions for this PCR were as follows: 1 × 98°C for 30 s; 10 × (98°C for 10 s, 60°C for 20 s and 72°C for 20 s) and 1 × 72°C for 2 min. The final product was purified with AMPure Beads (Beckman Coulter, California, United States) and verified and quantified with Bioanalyzer (Agilent, California, United States).

#### Sequencing and obtaining ZOTUs

The total ampoules were sequenced in team MiSeq Illumina (Illumina, California, United States), following instructions from the manufacturer, in a race pair end 2 × 300 using “MiSeq reagent kit v3 600 cycles” (Illumina, California, United States). Preprocessing and quality control were carried out using FastQC (Callahan et al., 2017), visualizing with MultiQC (Edgar, 2010) to eliminate low-quality sequences. Subsequently, the sequences were cleaned, filtering and eliminating those pairs presented too many differences or did not adequately overlap. We proceeded to group all the remaining sequencing reads for establishing ZOTU, determining which were unique and ordering them according to their total abundance. The chimera sequences and possible artifact sequences were then eliminated using the UCHIME algorithm (Andrews, 2010; Ewels et al., 2016). Once the “nonchimeric” sequences were obtained, the clusterization of ZOTU was performed using the USEARCH-UNOISE3 algorithm (Edgar et al., 2011; Edgar, 2018) with 99% identity for the generation of sequence clusters, aiming at the reconstruction of exact biological sequences. Finally, the ZOTU table was created, which collects the count of each ZOTU found per sample. With the ZOTU sequences detected, they were specifically aligned using the USEARCH U-LOCAL (Edgar, 2016a) algorithm in two nested rounds. The first specific round was aligned against a database, developed expressly for this study, of possible recurrent pathogenic bacteria in this type of samples, with a 99% alignment percentage. A second round was conducted with the taxa that remained unallocated in the first round; a specific alignment was made, but with the RDP database (Edgar, 2016b) and 90% identity. Finally, those ZOTU that were left without taxonomic assignment were classified according to the GreenGenes database v13 5 (Thermo Fisher, Madrid, Spain; Edgar, 2017), using the USEARCH predictor algorithm SINTAX (Chelius and Triplett, 2001). A cutoff point was applied based on the identity of this allocation relationship, fixed at a minimum of 97% identity, so that it was filtered to the taxonomic level at which this degree of identity was exceeded.

### Indices of diversity

The following indices were used to calculate alpha diversity: Simpson’s index (D; Simpson, 1949), Shannon index (H; Shannon, 2001), Pielou index (J; Pielou, 1969) and phylogenetic diversity index (PD; Faith, 1992).

### Biological assays on *Medicago sativa* (alfalfa) model

#### Determination of the test conditions of the ORGAON^®^ fertilizer

The optimal concentration of the ORGAON^®^ fertilizer was determined for the germination of alfalfa seeds (Medicago sativa L.) under controlled laboratory conditions. Serial dilutions (v/v) 1/1, 1/10, 1/64, 1/100, 1/128, 1/256, 1/512 and 1/1000 (mL ORGAON^®^.ml^–1^ / mineral water) were used. For the germination test, sterile 140 mm Petri dishes were used as containers, in which sterile filter paper (UV light 30 min) was placed on the base. Once sterilized, the filter paper discs were saturated with 4.5 ml of each dilution (experimental volume), avoiding the accumulation of excess liquid. A plate was used for each dilution of ORGAON^®^ fertilizer (Biaqui, Almería, Spain). A total of 160 alfalfa seeds were added for each treatment and evenly distributed using sterile tongs. Plates were then closed with a superimposed lid (under aerobic conditions, avoiding contamination by aerial microorganisms), and left in darkness at controlled temperature (22 ± 2°C). Each condition was assayed in triplicate. Germinated seeds (denoted by visible emerging radicle) were daily checked.

#### Sterilization of ORGAON^®^ fertilizer

For the control, the ORGAON^®^ (1/512) was sterilized using the same procedure described in section 4.2.2.

#### Improvement of ORGAON^®^ fertilizer with the incorporation of PGPB strains: Biofertilizer synthesis

The bacterial strains tested in this article were selected from previous studies, in which they were shown to be PGPB (Table 5).

**Table 5:** Information of the PGPB strains (Robas et al., 2021).

Name of the strain	Origin of isolation	AIA (μg.ml^−1^)	ACCd (+/−)	Siderophores (+/−)
*Pseudomonas agronomica*	*Medicago sativa*	5.85 ± 0.12	+	+
*Bacillus pretiosus*	*Medicago sativa*	5.61 ± 0.26	+	+

For enrichment of the ORGAON^®^ fertilizer with the proposed PGPB bacterial strains, the inoculum was standardized by making a bacterial suspension in 0.45% sterile saline solution with a final optical density of bacteria equivalent to 0.5 on the Mc Farland scale. 3 ml of these suspensions were added to 3 ml of a prepared 1/256 ORGAON^®^ dilution, achieving a final 1/512 dilution of the fertilizer.

#### Inoculation of the seeds and irrigation conditions

The preparation of the germination and plant growth tests were identical. A total of 100 seeds per treatment were sown in pots with a bottom of 2 cm^3^ of sterile river sand and 3 cm^3^ of agricultural soil. In total, 12 treatments were carried out, with three replicates each. Three types of irrigation were used, Chem-F, non-sterile ORGAON^®^ and sterile ORGAON^®^ (ORGAON^®^_ST). Each type of irrigation was enriched by adding the proposed bacteria: *Pseudomonas agronomica*, *Bacillus pretiosus*, the consortium of both, and an inoculum-free control. Each pot had an initial irrigation of 150 ml of treatment up to field capacity. Subsequently, it was irrigated four times with an experimental volume of 50 ml each.

#### Germination and plant growth conditions

The first days of germination, the pots were in natural light conditions. Once the seeds began to germinate, the pots were transferred to a programmed phytotron with a photoperiod of 11 h of light, light intensity of 505 μmoles.m^−2^.s^−1^ and a temperature range of 15–30°C.

#### Dry weight determination

Six weeks after the start of the trial, the seedlings were harvested. The collected biomass was dried at room temperature for 2 days. It was then weighed.

#### Statistical analysis

Once the null hypothesis of equality of means (ANOVA) had been rejected, to verify the existence of statistically significant differences between the different concentrations of ORGAON^®^, the post-hoc multiple comparison test of Duncan was performed. Differences were considered statistically significant when *p* < 0.05. To study the existence of significant differences between seeds irrigated with the different treatments, ORGAON^®^ fertilizer, sterilized ORGAON^®^ fertilizer, and the traditional Chem-F irrigation, the nonparametric Kruskal–Wallis test (Mann and Whitney, 1947; *N* < 30) was carried out. Differences were considered statistically significant when *p* < 0.05. To verify the effect of each treatment and the incorporation of the PGPB strains, an ANOVA was carried out. Rejecting the null hypothesis of mean equality, a pairwise comparison was carried out using Duncan’s *post-hoc* statistic. Differences were considered statistically significant when *p* < 0.05. The statistical package SPSS (Version 27.0, IBM Corp.) was used for all analyses.

## Conclusion

In view of the results obtained, the following conclusions can be drawn:

1. The fertilizer can be used in biosafety conditions. The metagenomic study of 16S rRNA amplicons reveals the absence of pathogens and phytopathogens.
2. Irrigation with ORGAON^®^ fertilizer (*v*/*v*; 1/512) results in a significant germination increase, 16% over regular Chem-F irrigation.
3. The maximum germination rate was reached at dilution 1/512 (*v*_Fert ORGAON®_/*v*_mineral water_) 144 h after sowing. Under these conditions, the germination rate was 46.88%.
4. The addition of the bacterial strain *Pseudomonas agronomica* or *Bacillus pretiosus* in the fertilizer promotes an increase in germination, biomass, and viability of *Medicago sativa* var. *Aragón.*

## Data availability statement

The data presented in the study are deposited in the BioProyect repository, accession number PRJNA906533.

## Author contributions

VF, MR, AP, and PJ: conceptualization, methodology, formal analysis, and writing—review and editing. VF, MR, and PJ: software and investigation. AP: validation and data curation. AP and PJ: resources, supervision, project administration, and funding acquisition. VF and MR: writing—original draft preparation and visualization. All authors contributed to the article and approved the submitted version.

## Funding

This research has been funded by FUNDACIÓN UNIVERSITARIA SAN PABLO CEU (Microbiology SAI) and BANCOs SANTANDER, grant number FUSP-BS-PPC01/2014. Likewise, this project has been financed with the European Funds oriented to the ecological transition and digital transition, of the national plan for scientific, technical and innovation research 2021-2023, within the framework of the recovery, transformation and resilience plan. File number TED2021-132285A-I00.

## Conflict of interest

The authors declare that the research was conducted in the absence of any commercial or financial relationships that could be construed as a potential conflict of interest.

## Publisher’s note

All claims expressed in this article are solely those of the authors and do not necessarily represent those of their affiliated organizations, or those of the publisher, the editors and the reviewers. Any product that may be evaluated in this article, or claim that may be made by its manufacturer, is not guaranteed or endorsed by the publisher.
