# Supplementary material for: Valorization as a biofertilizer of an agricultural residue leachate: Metagenomic characterization and growth promotion test by PGPB in the forage plant Medicago sativa (alfalfa)
Source: Front Microbiol. 2022 Dec 22;13:1048154. doi: 10.3389/fmicb.2022.1048154 (PMC9815802; doi:10.3389/fmicb.2022.1048154)
Supplement: Supplementary file 1 [file Table_1.DOCX]

Supplementary Material

# Supplementary Data

**Table S1**: Taxonomic categories found in the ORGAON® fertilizer.

| FILUM | CLASE | ORDEN | FAMILIA | GENERO | ESPECIE |
| --- | --- | --- | --- | --- | --- |
| *Euryarchaeota* | *Methanomicrobia* | *Methanosarcinales* | *Methanotrichaceae* | *Methanothrix*  *0,38%*  ±  *0,38%* | *Methanothrix*  *harundinacea* |
| *Firmicutes* | *Clostridia* | *Clostridiales* | *Clostridiales Incertae Sedis XI* | *Unclassified*  *13,91%* | *Unclassified* |
|  |  |  | *Clostridiaceae* | *Clostridium*  *3,34%*  ±  *0,07%* | *Clostridium*  *acetireducens* |
|  |  |  |  |  | *Unclassified* |
|  |  |  | *Clostridiaceae 2* | *Alkaliphilus*  *9,79%*  ±  *0,16%* | *Alkaliphilus*  *oremlandii* |
|  |  |  |  |  | *Alkaliphilus*  *halophilus* |
|  |  |  | *Clostridiaceae 1* | *Unclassified*  *0,12%* | *Unclassified* |
|  |  |  | *Eubacteriaceae* | *Garciella*  *1,22%*  ±  *0,05%* | *Garciella*  *Nitratireducens* |
|  |  |  |  | *Acetobacterium*  *0,39%*  ±  *0,07%* | *Acetobacterium*  *woodi* |
|  |  |  | *Peptococcaceae* | *Desulfitibacter*  *0,59%*  ±  *0,09%* | *Desulfitibacter*  *alkalitolerans* |
|  |  |  | *Hungateiclostridiaceae* | *Saccharofermentans*  *0,24%*  ±  *0,24%* | *Saccharofermentans*  *acetigenes* |
|  |  |  | *Ruminococcaceae* | *Unclassified*  *0,24%* | *Unclassified* |
|  |  | *Thermoanaerobacterales* | *Unclassified* | *Unclassified*  *0,14%* | *Unclassified* |
|  |  | *Unclassified* | *Unclassified* | *Unclassified*  *2,75%* | *Unclassified* |
|  | *Bacilli* | *Bacillales* | *Sporolactobacillae* | *Unclassified*  *2,80%* | *Unclassified* |
|  |  |  | *Bacillaceae 1* | *Bacillus*  *3,23%*  ±  *0,17%* | *Bacillus*  *shackletonii* |
|  |  |  |  |  | *Bacillus*  *cellulosilyticus* |
|  |  |  | *Bacillaceae 2* | *Vulcanibacillus*  *0,39%*  ±  *0,39%* | *Vulcanibacillus*  *modesticaldus* |
|  |  |  | *Unclassified* | *Unclassified*  *2,79%* | *Unclassified* |
|  |  | *Lactobacillales* | *Aerococcaceae* | *Aerococcus*  *0,25%* | *Aerococcus*  *urinaeequi* |
|  |  |  |  | *Facklamia*  *0,18%*  ±  *0,18%* | *Facklamia*  *tabacinasalis* |
|  |  |  | *Lactobacillae* | *Unclassified*  *0,25%* | *Unclassified* |
|  |  |  |  | *Lactobacillus*  *0,38%*  ±  *0,38%* | *Lactobacillus*  *amylotrophicus* |
|  | *Erysipelotrichi* | *Erysipelotrichales* | *Erysipelotrichaceae* | *Erysipelothrix*  *0,17%*  ±  *0,17%* | *Erysipelothrix*  *rhusiopathiae* |
|  | *Tissierellia* | *Tissierellales* | *Tissierellaceae* | *Tissierella*  *11,77*  ±  *0,18%* | *Tissierella*  *creatinophila* |
|  |  |  |  |  | *Tissierella*  *carlieri* |
|  |  |  |  |  | *Unclassified* |
|  |  |  |  | *Tepidimicrobium*  *0,36*  ±  *0,36%* | *Tepidimicrobium*  *xylanilyticum* |
|  |  |  |  | *Soehngenia*  *0,15*  ±  *0,15%* | *Soehngenia*  *saccharolytica* |
|  |  |  | *Peptoniphilaceae* | *Anaerococcus*  *0,43*  ±  *0,43%* | *Anaerococcus*  *octavius* |
|  |  | *Unclassified* | *Unclassified* | *Sedimentibacter*  *0,93*  ±  *0,93%* | *Sedimentibacter*  *hydroxybenzoicus* |
|  |  |  |  | *Dethiosulfatibacter*  *0,34*  ±  *0,34%* | *Dethiosulfatibacter*  *Aminovorans* |
| *Proteobacterias* | $\alpha$*-proteobacteria* | *Rhodobacterales* | *Rhodobacteraceae* | *Amaricoccus*  *25,72*  ±  *0,05%* | *Amaricoccus*  *kaplicensis* |
|  |  |  |  |  | *Unclassified* |
|  | $\gamma$*-proteobacteria* | *Pseudomonadales* | *Pseudomonadaceae* | *Pseudomonas*  *0,66*  ±  *0,07%* | *Pseudomonas*  *otitidis* |
|  |  |  |  |  | *Unclassified* |
|  |  | *Oceanospirillales* | *Halomonadaceae* | *Halomonas*  *0,61*  ±  *0,61%* | *Halomonas*  *sulfidaeris* |
|  |  |  |  |  | *Halomonas*  *mongoliensis* |
|  | $\delta$*-proteobacteria* | *Desulfuromonadales* | *Desulfuromonadaceae* | *Desulfuromonas*  *1,21*  ±  *0,09%* | *Desulfuromonas*  *svalbardensis* |
|  | $\beta$*-proteobacteria* | *Burkholderiales* | *Alcaligenaceae* | *Oligella*  *0,80*  ±  *0,05%* | *Oligella*  *Urethralis* |
| *Bacteroidetes* | *Bacteroidia* | *Bacteroidales* | *Porphyromonadacceae* | *Petrimonas*  *14,97%*  ±  *0,13%* | *Petrimonas*  *sulfuriphila* |
|  |  |  |  | *Unclassified*  *2,8%* | *Unclassified* |
|  |  |  | *Rikenellaceae* | *Unclassified*  *4,21%* | *Unclassified* |
|  |  |  | *Dysgonamonadaceae* | *Proteiniphilum*  *2,80%*  ±  *0,08%* | *Proteiniphilum*  *acetatigenes* |
|  |  |  | *Unclassified* | *Unclassified*  *1,21%* | *Unclassified* |
|  |  | *Unclassified* | *Unclassified* | *Unclassified*  *0,61%* | *Unclassified* |
|  | *Unclassified* | *Unclassified* | *Unclassified* | *Unclassified*  *0,28%* | *Unclassified* |
| *Tenericutes* | *Mollicutes* | *Acholeplasmatales* | *Acholeplasmataceae* | *Acholeplasmaç*  *1,82*  ±  *0,07%* | *Acholeplasma*  *parvum* |
|  |  |  |  |  | *Acholeplasma*  *palmae* |
|  |  |  |  | *Unclassified*  *1,96%* | *Unclassified* |
| *Thermotogae* | *Thermotogae* | *Petrogales* | *Petrogaceae* | *Defluviitoga*  *0,59%*  ±  *0,59%* | *Defluviitoga*  *tunisiensis* |
| *Spirochaetes* | *Unclassified* | *Unclassified* | *Unclassified* | *Unclassified*  *0,54*  ±  *0,54%* | *Unclassified* |
| *Actinobacteria* | *Actinobacteria* | *Actinomycetales* | *Unclassified* | *Unclassified*  *0,24%* | *Unclassified* |
|  |  | *Propionibacteriales* | *Propionibacteriaceae* | *Tessaracoccus*  *0,24%*  ±  *0,24%* | *Tessaracoccus*  *oleiagri* |
